# Uptake and Retention of Laser-synthesized Boron Nanoparticles in Tumor Cells and Fibroblasts

**DOI:** 10.1134/S1607672925601775

**Published:** 2026-04-20

**Authors:** A. I. Kasatova, K. S. Kuzmina, P. A. Kotelnikova, E. V. Barmina, K. O. Aiyyzhy, A. A. Laktionov, D. I. Tselikov, I. V. Sozaev, I. V. Lunyov, S. M. Klimentov, M. S. Grigoryeva, D. S. Petrunya, E. L. Zavjalov, S. Yu. Taskaev, S. M. Deyev, I. N. Zavestovskaya

**Affiliations:** 1 Lebedev Physical Institute, Russian Academy of Sciences, Moscow, Russia; 2 Budker Institute of Nuclear Physics, Siberian Branch, Russian Academy of Sciences, Novosibirsk, Russia; 3 Novosibirsk State University, Novosibirsk, Russia; 4 Shemyakin‒Ovchinnikov Institute of Bioorganic Chemistry, Russian Academy of Sciences, Moscow, Russia; 5 Prokhorov General Physics Institute, Russian Academy of Sciences, Moscow, Russia; 6 National Research Nuclear University MEPhI, Engineering Physics Institute of Biomedicine, Moscow, Russia; 7 National Research Center Kurchatov Institute, Moscow, Russia; 8 Institute of Cytology and Genetics, Siberian Branch, Russian Academy of Sciences, Novosibirsk, Russia; 9 National Research Ogarev Mordovia State University, Saransk, Russia

**Keywords:** boron neutron capture therapy, nanoparticles, laser synthesis, cell lines, cytotoxicity, atomic emission spectrometry

## Abstract

Boron Neutron Capture Therapy (BNCT) is one of the innovative methods for treating oncological diseases. Its selectivity is based on the targeted delivery of the boron-10 isotope to tumor cells, followed by neutron irradiation, the ^10^B(n, α)^7^Li reaction occurs with a local release of 2.79 MeV of energy. Budding boron delivery agents are nanoscale systems. This study evaluated in vitro cytotoxicity, accumulation, and retention of elemental boron nanoparticles, synthesized by laser ablation and laser fragmentation, in U87 and BT474 tumor cells and BJ-5ta fibroblasts. It was shown that both types of nanoparticles exhibit low cytotoxicity at therapeutically relevant concentrations. Boron accumulation was maximal after 24 h of incubation and was significantly higher in tumor cells, especially in the BT474 cell line, compared to fibroblasts. The obtained data indicate the promise of these nanoparticles as boron delivery agents for BNCT.

## INTRODUCTION

The global incidence of malignant neoplasms continues to rise, with projections estimating a 77% increase in new cases by 2050 compared to 2022, reaching 35 million annually [1]. Oncological diseases remain the second leading cause of death, including in Russia [2], and achieving significant breakthroughs requires extensive efforts in prevention and therapeutic innovation. This alarming trend underscores the need for novel treatment methods, among which Boron Neutron Capture Therapy (BNCT) is a promising approach. This method represents a binary type of radiation treatment. Its selectivity is achieved through the ability of the boron-10 isotope, accumulated in the tumor, to capture thermal neutrons, initiating a subsequent localized nuclear reaction. The products of this reaction—alpha particles and lithium nuclei—possess high linear energy transfer and an extremely short range, comparable to the size of a single cell. This ensures the precise destruction of only those cells containing boron, inflicting minimal damage to surrounding healthy tissues. A key condition for the successful application of BNCT is the development of compounds capable of ensuring highly selective delivery of boron to the tumor. To date, the drug borophenylalanine (BPA) [3–5] is used in clinical practice, and sodium borocaptate (BSH) has also been used in previous clinical trials. Despite encouraging clinical trial results, variability in boron-10 accumulation and rapid excretion from the body, necessitating continuous drug infusion even during irradiation, highlight the need to develop more efficient and selective boron delivery agents. In this regard, the field of nanoscale delivery systems is actively advancing [6–11]. This study investigates elemental boron nanoparticles, enriched to 85% with the boron-10 isotope, obtained via laser synthesis and functionalized with a biocompatible Silane-PEG coating to improve their pharmacokinetic properties. Boron content up to 100% of the particle mass, of which 85% is the isotope required for the reaction, combined with the high packing density of atoms, allows for minimizing the administered dose to achieve therapeutic concentration and reducing the overall toxic load. Unlike highly stable boron carbide, elemental boron in an aqueous environment can slowly oxidize into soluble compounds and be excreted from the body.

The aim of the study was to determine the cytotoxicity, accumulation, and retention of boron in cells following the administration of the investigated nanoparticles.

## MATERIALS AND METHODS

Boron nanoparticles (NPs) were obtained using the method of nanosecond laser fragmentation (LF NPs) of boron-10 micropowder in isopropanol in a flow cell, as well as by the method of femtosecond laser ablation (LA NPs) in acetonitrile. The experimental setups, materials, methods, and results are described in more detail in [6, 9].

**Nanoparticle surface modification.** Surface functionalization of boron nanoparticles with polyethylene glycol (PEG) was performed using a modified Stöber method [11]. A mixture containing 1 mg of nanoparticles in 1 ml of ethanol was combined with 65 µl of water, 20 µl of 30% ammonium hydroxide, and 100 µl of PEG-silane solution (1 g/L) in ethanol. The mixture was sonicated and incubated at 60°C for 2 h, followed by overnight incubation at 25°C. The resulting B-PEG nanoparticles were centrifuged three times (20 000 g, 15 min) and washed with ethanol and deionized water.

**Determination of the chemical composition of nanoparticles.** The analysis was performed by inductively coupled plasma mass spectrometry (ICP-MS) after dissolving the samples in aqua regia for 2 h with heating to 80°C and for 24 h at 25°C. To determine the boron concentration, a boron calibration solution (Sigma) was used, assuming 20% B-10 and 80% B-11. To determine the zirconium concentration, calibration solutions prepared from zirconium chloride ZrCl4 (Sigma Aldrich) were used. To determine the concentration of Ni, Cu, Mn, Zn, the Instrument Calibration Standard 2 solution (Perkin Elmer) was used. A 10% aqua regia solution (nitric and hydrochloric acids—Sigma Tech) was used for the baseline and for diluting the calibration solutions.

**Cell culture.** The study utilized three human cell lines: BJ-5ta fibroblasts, BT-474 breast carcinoma cells (Lobachevsky State University of Nizhny Novgorod, Russia), and U87 glioblastoma cells (Institute of Cytology and Genetics SB RAS, Novosibirsk, Russia). Cell lines were cultured in DMEM/F-12 medium (Biolot, Russia) supplemented with 10% fetal bovine serum (FBS, Dia-M, Russia) and 50 mg/ml gentamicin (Dalhimpharm, Russia) at 37°C and 5% CO_2_. The BT-474 line was cultured in RPMI medium (Biolot, Russia) under the same conditions.

**Cytotoxicity assessment of nanoparticles.** Cytotoxicity of elemental boron nanoparticles was evaluated using the MTT assay. Cells in the exponential growth phase were seeded into 96-well plates at a density of 1 × 10^4^ cells per well. Nanoparticles diluted in culture medium over a wide range of boron-10 concentrations were added and incubated for 24 h. Control wells were incubated in medium without boron-10. Subsequently, MTT solution was added to each well. The optical density of the solutions in each well was measured using a Multiskan SkyHigh spectrophotometer (Thermo Fisher Scientific, USA) at a wavelength of 595 nm. The percentage of viable cells was calculated relative to the control.

**Boron uptake and retention assessment.** Boron concentration in cells was determined using inductively coupled plasma atomic emission spectrometry (ICP-AES). During the exponential growth phase, the medium was replaced with fresh medium containing nanoparticles at a boron-10 concentration of 40 µg/ml in complete culture medium, followed by incubation for 24 h. The cells were then washed twice with sodium phosphate buffer, detached using a trypsin-Versene solution, and counted in each sample using an automated Countess Cell Counter (Invitrogen), followed by pelleting via centrifugation. Sample preparation involved digestion with concentrated nitric acid (analytical grade, 69%, Panreac AppliChem) at 90°C. Boron concentration measurements were performed using an ICPE-9820 spectrometer (Shimadzu, Japan). The final intracellular boron concentration was calculated using the formula: measured concentration × sample volume / (cell count × 10^6^).

**Statistical analysis.** Data were statistically processed using STATISTICA 10.0 software, employing the nonparametric Mann–Whitney U-test. Results are presented as mean ± standard deviation (*M* ± SD). Differences were considered statistically significant at *p* < 0.05.

## RESULTS

**Fig. 1.**
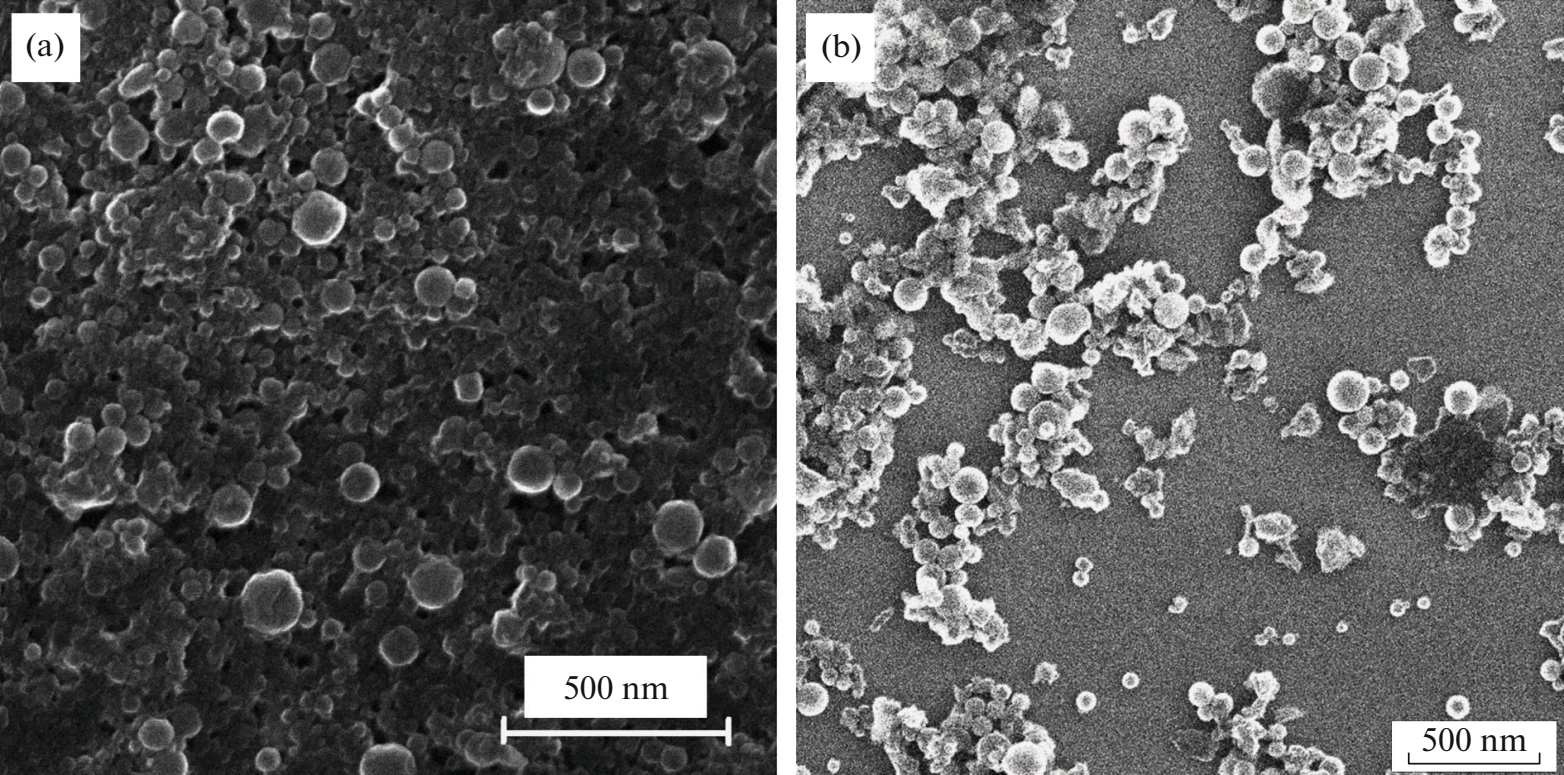
SEM images of synthesized boron nanoparticles: (a) after laser ablation (LA), (b) after laser fragmentation (LF).

Scanning electron microscopy (SEM) analysis revealed that LA NPs (laser ablation nanoparticles) possess a spherical shape. Their physical size distribution followed a log-normal pattern with a modal diameter of 37 nm (Fig. 1a).

Dynamic light scattering analysis showed that laser fragmentation leads to the formation of boron nanoparticles ranging in size from 40 to 200 nm. SEM microscopy of the supernatant after centrifugation indicated an average nanoparticle size of 50 nm (Fig. 1b).

**Fig. 2.**
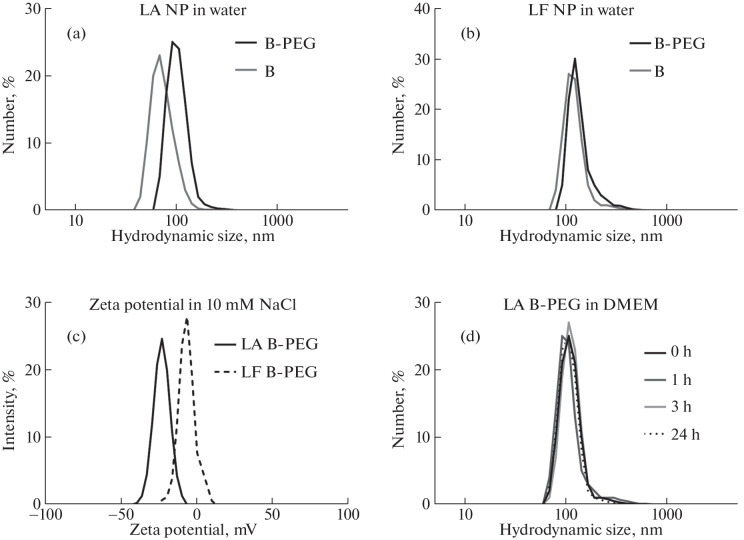
Hydrodynamic size of LA (a) and LF (b) boron nanoparticles in water, before and after PEG coating. Zeta potential of B-PEG nanoparticles (c). Stability of B-PEG nanoparticles in culture medium (d).

After coating LA NPs with polyethylene glycol (PEG), their hydrodynamic diameter in water increased from (65 ± 20) to (104 ± 29) nm. The zeta potential of the coated LA boron NPs was (–29 ± 5) mV (Figs. 2a, 2c).

After coating LF NPs (laser fragmentation nanoparticles) with PEG, their hydrodynamic diameter in water increased from (121 ± 42) to (141 ± 52) nm. The zeta potential of the coated LF boron NPs was (–6.3 ± 3.4) mV (Figs. 2b, 2c).

According to the ICP-MS results, both types of nanoparticles contained boron in their composition, enriched with the boron-10 isotope up to 85%; the concentration of impurities is presented in Table 1.

**Table 1.** ICP-MS results for two types of NPs, data are presented in %

	**LA NP**	**LF NP**
**B-10**	84.2 ± 7.0	67.4 ± 4.6
**B-11**	13.4 ± 1.5	11.0 ± 0.5
**Cu**	1.2 ± 0.1	0.9 ± 0.1
**Ni**	1.0 ± 0.1	1.5 ± 0.1
**Mn**	0.2 ± 0.1	0.2 ± 0.1
**Zn**	0.1 ± 0.2	0.4 ± 0.2
**Zr**	0.0 ± 0.1	18.5 ± 1.7

PEG-coated NPs maintained colloidal stability in water for at least 1 month. In culture medium supplemented with 10% serum, the particles remained stable for a minimum of 24 h. The hydrodynamic diameter of LA NPs immediately, and after 1, 2, and 24 h of exposure to the medium was (115 ± 38), (116 ± 52), (117 ± 38), and (118 ± 32) nm, respectively. The hydrodynamic diameter of LF NPs under the same conditions was (145 ± 66), (147 ± 59), (148 ± 56), and (145 ± 66) nm, respectively; no further measurements were taken (Fig. 2d).

**Fig. 3.**
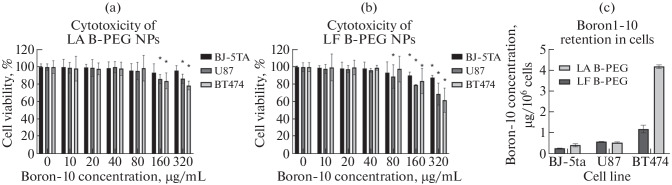
Cytotoxicity of elemental boron nanoparticles synthesized by LF (a) and LA (b) methods as a function of boron-10 concentration. Boron-10 uptake by cell lines after 24-h incubation with LA and LF boron nanoparticles (c).

Initial experiments with high nanoparticle doses demonstrated that both NP types, at boron-10 concentrations up to 80 µg/ml, did not cause significant reduction in cell viability (Figs. 3a, 3b), indicating their safe use at concentrations required for successful BNCT.

For all three cell lines, boron accumulation was maximal after 24 h of incubation (Table 2). Since the ICP-AES method cannot determine isotopic composition, Table 2 presents data for total boron. Data for the 24-h time point, recalculated based on the boron-10 content in the nanoparticles, are presented as a diagram in Fig. 3c.

**Table 2.** Boron content in cell lines depending on incubation time and laser synthesis method

NP type	Time point (h)	Cell line		
		BJ-5ta	U87	BT474
**LF**	1	0.02 ± 0.004	0.31 ± 0.04	Below detection limit
	2	0.09 ± 0.013	0.4 ± 0.01	Below detection limit
	24	0.33 ± 0.006	0.74 ± 0.01	1.84 ± 0.01
**LA**	1	0.41 ± 0.07	0.33 ± 0.03	0.49 ± 0.05
	2	0.39 ± 0.02	0.37 ± 0.02	0.5 ± 0.03
	24	0.63 ± 0.03	0.7 ± 0.01	5.23 ± 0.74

**Fig. 4.**
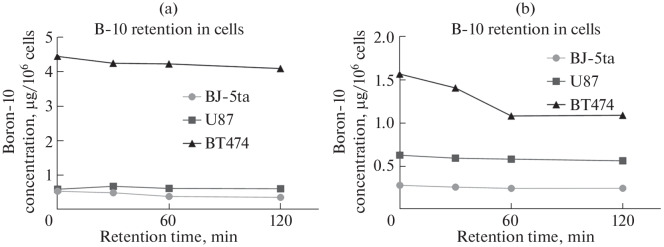
Accumulation and retention of boron-10 in normal and tumor cell lines after 24-h incubation with LA (a) and LF (b) boron nanoparticles.

Boron retention in cells was also assessed at 30, 60, and 120 min after removing the nanoparticle-containing medium following a 24-h incubation (Fig. 4). Boron accumulation was lowest in fibroblasts compared to the tumor cell lines: the boron-10 concentration after 24 h of incubation with LF NPs was 0.28 µg/10^6^ cells. The boron content in cells gradually decreased, reaching 0.24 µg ^10^B/10^6^ cells (Fig. 4b). The same trend was observed for LA NPs: boron content immediately after removal of the NP-containing medium was 0.54 µg/10^6^ cells. Over 120 min, the boron content decreased to 0.35 µg/10^6^ cells (Fig. 4a). The boron-10 content in U87 cells after incubation with both NP types was approximately equal, around 0.6 µg/10^6^ cells (Fig. 3c). Relatively prolonged boron retention was observed in cells: over 120 min, the intracellular concentration decreased by less than 0.1 µg/10^6^ cells (Fig. 4). The highest boron concentration was found in BT474 cells after 24 h of incubation, reaching 4.4 and 1.56 µg/10^6^ cells for LA and LF NPs, respectively (Fig. 3c). Cells pre-incubated with LF NPs demonstrated the most rapid boron loss (Fig. 4b). Over 120 min, the boron content decreased by 0.5 µg/10^6^ cells. In the case of LA NPs, boron retention was more prolonged (Fig. 4a).

## DISCUSSION

Elemental boron nanoparticles are considered an efficient delivery system for boron-10 in BNCT due to their high number of boron atoms per particle [12]. Approved clinical drugs, BPA and BSH, contain one and twelve boron-10 atoms per molecule, respectively, whereas elemental boron nanoparticles can deliver thousands of boron atoms into a cell simultaneously [12]. The transport mechanism of borophenylalanine into tumor cells is mediated by L-amino acid transporters (LAT1, LAT2, ATB(0, +)), which actively transport L-amino acids across cell membranes during active tumor growth [13]. In contrast, sodium borocaptate accumulation in tumor cells occurs primarily via passive diffusion from the blood [14]. The transport mechanism for nanoparticles differs from that of molecular boron-containing drugs, as their cellular internalization occurs via endocytosis [15].

Terada et al. evaluated LAT-1 expression and boron uptake from borophenylalanine at a concentration of 20 µg ^10^B/ml in cervical cancer cell cultures *in vitro*. They found that uptake was independent of incubation time and ranged from 0.0437 to 0.092 µg ^10^B/10^6^ cells, depending on the cell line. Boron concentration dropped sharply within the first 30 min after replacing the medium with one free of borophenylalanine, with boron retention ranging from 11 to 23% over a 4-hour observation period [16].

Several more selective third-generation boron delivery agents are currently under development [17]. Tsurubuchi et al. assessed the intracellular uptake of a boron compound derived from α-d-mannopyranoside, MMT1242, compared to BPA. The study showed that boron from MMT1242 was taken up by mouse colon cancer CT26 cells at approximately twice the level of BPA (~0.32 vs. ~0.15 µg/10^6^ cells, respectively). The authors also noted that BPA content decreased to zero within 60 min after cell washing, while the intracellular concentration of MMT1242 remained around 0.3 µg/10^6^ cells throughout the observation period [18].

Laird et al. compared mesoporous silica-based nanoparticles with covalently attached sodium borocaptate (BSH-BPMO) with standard BSH and BPA. They demonstrated that the nanoparticles were efficiently taken up by cancer cells, with boron accumulation reaching 19.15% after 24 h of incubation, which is 58 times higher than the 0.33% observed for BPA. The researchers attribute such low values for BPA to less intense uptake or rapid exchange via the LAT1 amino acid transporter. BNCT using BSH-BPMO led to the destruction of tumor spheroids after neutron irradiation, whereas standard BSH and BPA drugs showed significantly lower efficacy [19].

An obvious advantage of elemental boron nanoparticles is that boron constitutes 100% of the particle’s mass. In contrast, boron nitride (BN) nanoparticles contain only 44% boron by mass, and boron carbide (B_4_C) nanoparticles contain 78%.

Our experiments revealed that cells pre-incubated with LA NPs accumulate boron to a greater extent than with LF NPs. This may be due to the difference in the charge of these nanoparticles, whose zeta potentials were –29 mV and –6 mV for LA NPs and LF NPs, respectively. Nishikawa et al. also suggest that efficient cellular uptake of nanoparticles is facilitated by their negative charge [20].
